# Practice Versus Precept

**Published:** 1849-03

**Authors:** 


*Practice versus Precept.*—Attempts are frequently made by different persons to impair public confidence in medical science. Various motives are involved in such efforts. Some are actuated by cupidity, others, with more honesty, but less good sense, become infatuated with a popular medical delusion. In many instances of the latter class of individuals, the aberration is transient, more especially if the persons have the good fortune not to become very deeply committed, and still more, if they have not, like Dr. Sangrado, written a book, in which case they are apt to find themselves in an awkward position. Some striking exemplifications of the antithesis of precept and practice, are furnished in the history of some of these self-sufficient belligerents. A case in point, which has suggested these remarks, has happened recently to fall under our notice in the pages of a weekly newspaper published in a New England village, the *Northampton Courier.* Dr. Sylvester Graham (appropriating the title of the profession, which he holds in so low estimation,) is a resident of that village. Many of our readers, probably, have heard of him, as he has rendered himself notorious by sundry publications, and by lecturing upon the bran bread system of diet. We give the article taken from the paper referred to, in full. The editor's remarks will commend themselves as sensible, and appropriate to the occasion:—

*Dr. Graham's Advice.*—An article from the prolific pen of Dr. Sylvester Graham, giving advice on diet and regimen, to California adventurers, appeared in the Gazette last week, with a sort of indefinite commendation by the editor. The Doctor is a good temperance man, and gives wholesome advice on that subject. His advice in regard to clothing, diet and bathing, contains more or less good doctrine, mixed up with notions of his own, of which we express no opinion. But his attempt to dissuade from the use of medicine, even in the most dangerous sickness, is, in our judgment, wrong and dangerous, and is contradicted by his practice in relation to himself. He says:—

"If you become ill, or even decidedly sick, I charge you, nay, I solemnly conjure you, *take no medicine.* Let no one prevail on you—let nothing induce you to swallow drugs, even if the Asiatic cholera attacks you. In all cases of indisposition, in all kinds of sickness, let cold water, pure air, abstemiousness, fasting, hand-rubbing, and rest, constitute your remedial means; and let your administration of these means correspond, as to promptitude, energy, freedom and boldness, with the force of your morbid symptoms; and, if it be not your destiny to die in spite of any means, you wili soon recover and be on your feet again.

If you wisely observe the regimen I have prescribed for you, there is hardly a probability that you will be sick."

Now mark the Doctor's practice, and see how it agrees with his teachings. A few years ago he was dangerously sick, and employed a regular physician to attend him, submitting himself entirely to his advice and prescriptions. He took medicine regularly, and that which contained opium and calomel, knowing that they were ingredients of what he took, though he had often scouted the use of those drugs. It was when death stared him in the face that he abandoned his own system; and it is now, when in health, that he counsels others to adhere to it, at all hazards.

As he claims to be "*a public man*," and gives advice with the confidence of a learned and accredited practitioner, it becomes him to show why he advises those who go to California, to "*throw physic to the dogs*," when he uses it himself in dangerous sickness.

The above is by no means a rare illustration of a similar contrast between precept and practice. We have ourselves met with not a few cases of like character. But, so far as our observation goes, the instances are very few in which a person whose *philanthropy* and *sense of duty* constrained him to employ great diligence in advocating an imposition, or delusion, so long as his infatuation lasted, feels it incumbent to take equal pains to undo the mischief he may possibly have done, when, afterward, his sober reflections, or experience, have led him to perceive the errors he had fallen into. This is a significant fact.

